# The Locus Coeruleus- Norepinephrine System in Stress and Arousal: Unraveling Historical, Current, and Future Perspectives

**DOI:** 10.3389/fpsyt.2020.601519

**Published:** 2021-01-27

**Authors:** Jennifer A. Ross, Elisabeth J. Van Bockstaele

**Affiliations:** Department of Pharmacology and Physiology, College of Medicine, Drexel University, Philadelphia, PA, United States

**Keywords:** arousal, norepinephrine, locus coeruleus, electroencephalography, transmission electron microscopy

## Abstract

Arousal may be understood on a spectrum, with excessive sleepiness, cognitive dysfunction, and inattention on one side, a wakeful state in the middle, and hypervigilance, panic, and psychosis on the other side. However, historically, the concepts of arousal and stress have been challenging to define as measurable experimental variables. Divergent efforts to study these subjects have given rise to several disciplines, including neurobiology, neuroendocrinology, and cognitive neuroscience. We discuss technological advancements that chronologically led to our current understanding of the arousal system, focusing on the multifaceted nucleus locus coeruleus. We share our contemporary perspective and the hypotheses of others in the context of our current technological capabilities and future developments that will be required to move forward in this area of research.

## Introduction

The concept of arousal is one that is best defined on a spectrum. Through a clinical lens, behaviors relating to inattention or cognitive dysfunction associated with under-stimulation may reflect mild to moderate levels of an arousal deficiency, with excessive daytime sleepiness and sedation being symptoms of the most extreme cases. On the other hand, behavior associated with hypervigilance, insomnia, overstimulation, and fear or panic may indicate a mild or moderate level of excessive arousal, with psychosis and hallucinations being symptoms in the most extreme cases. In the center of the spectrum, arousal is a state of wakefulness in which an individual is awake, alert, able to create and problem solve (1). However, as a subject of investigation, arousal can be difficult to define, particularly when considering the dynamic neural processes that underlie this spectrum of brain states. For example, the brain state associated with overstimulation of arousal systems may describe a stressed brain state. Continued efforts to define stress and its relationship to arousal reflect a longstanding issue that has been described by others (2). Both have been characterized across scientific disciplines using a broad range of descriptors, many of which are nearly synonymous, making it challenging to converse across neuro-behavioral study fields.

Cognitive psychology, neurobiology, neuroendocrinology, and cognitive neuroscience have shared technological advancements expanding our understanding of the behavioral, network-, circuit- and synaptic-level responses to stimuli. However, silos of advancements within each discipline persist, continuing to hinder the potential for interdisciplinary synergism. In perspective shared by Pessoa, Pessoa describes a significant modern challenge in brain-behavior research embodied by a granular focus on “causal explanations” at the expense of theoretical and conceptual descriptions that may be shared and provide deeper understanding across disciplines (3).

In the sections that follow, we discuss the Locus Coeruleus (LC)- Norepinephrine (NE) system in the context of this quandary as it illustrates a system with interdisciplinary interest that has made significant strides in recent years and yet lacks a common vernacular to facilitate cross-disciplinary communication. The LC-NE system is involved in various neurobiological processes, including the modulation of sleep-wake cycles, facilitation of attention, responses to stress, initiation memory formation, and retrieval. It modulates blood flow, metabolism, and the distribution of oxygen and glucose throughout the brain. Thus, it is evident that the LC is a brain region that directly or indirectly has been investigated or described across several scientific disciplines. Though scarce, we discuss works in the literature that integrate the findings of these efforts across multiple levels of neurobiological function (4–8).

We start by providing historical context, first by the recollection of the neuroanatomical and physiological studies that led to our current understanding of the LC-NE system and subsequently discussing the evolution of Arousal Theory. Because the broader fields of cognitive psychology and neurobiology have historically advanced our understanding of arousal, primarily independently of each other, we first discuss them separately. We then transition to a synthesis of recent literature that brings to light a critical intersection of these fields by discussing current theories that directly or indirectly implicate the LC-NE system in large-scale network dynamics under various arousal states. Finally, we discuss pathology related to the LC-NE system dysfunction, emphasizing hyper-arousal in stress-related disorders. Along the way, we discuss technological advancements that preceded each milestone of conceptual evolution and the past and current day challenges that have emerged over time.

## The Study of Stress: A Historical Perspective

### The Origin of “Stress” and the Divergence of Its Scientific Study

Early scientific observations on how the body adapts to a life-threatening situation, aging, or disease, are the foundations of stress-related research as we know it today (9). The earliest scientific report of such an adaptation was published in 1914 by Walter Cannon and described the adrenal medullary response to pain, asphyxiation, and emotions such as fear and rage as an “emergency response.” He notes that the release of catecholamines norepinephrine and epinephrine produces 'striking bodily alterations' in which blood flow shifts away from the abdomen and toward the lungs, heart, limbs, and central nervous system (10). Cannon later conceptualized the sympathoadrenal system as a “mobilizer of bodily forces for struggle” that connected the adrenal medulla's endocrine functions with the sympathetic nervous system to coordinate physiological responses to crises. Thus, Cannon's observations founded the physiological basis for the 'fight or flight' response. Throughout the 1920s, Cannon published highly impactful work on regulating internal states and coined the term “homeostasis.” Homeostasis describes the intricate coordination of mechanisms between the autonomic nervous and endocrine systems to regulate physiological parameters first observed by Bernard (11). Bernard and Cannon's observations fundamentally contributed to the body's conceptualization as a self-regulating system that required a balance of physical and chemical states with the external environment. Importantly, Cannon brought to light the clinical impact of emotional distress, which he asserted could dysregulate these homeostatic mechanisms and generate disease.

In the decades that followed the initial conception of “stress,” scientists across disciplines debated how to define the stress response and measure it under experimental conditions (2). Some took a reductionist approach, derived from the concept of a body economy (12), that defined the body as an energy system from which elements of the environment would mobilize resources to respond physiologically or behaviorally (13, 14). Others emphasized the emotional aspects of stress (15, 16) in which emotions manage both motivational resources and regulate behavioral and cognitive activation as a preparatory step to formulating action (17, 18).

Meanwhile, other investigators refuted the idea of a unified explanation of such responses. They emphasized the diversity of stress effects (19), noting that sleep deprivation caused specific detriments while other stimuli such as noise or heat compromised other factors (2). Creating a single definition across disciplines proved an insurmountable task that left each field to define “stress” on its terms. One could certainly argue that divergence from the singular concept of “stress” in the scientific study was necessary to expand our understanding of a complex system. However, beneficial the departure was, it also created hindrances as silos of neurobiology advancements, neuroendocrinology, and cognitive neuroscience occurred in parallel, without synergism.

### The Discovery of the Hypothalamic-Pituitary-Adrenal Axis

Technological advancements facilitated continued growth in neurobiology and neuroendocrinology, particularly the monumental discovery of microelectrodes (20). Although primitive in their design, the creation of such electrodes by the “founding fathers of neurobiology” allowed for recording electrical impulses for the first time (21, 22). Techniques of this kind sparked the interest of Geoffrey Harris, credited as the founding father of neuroendocrinology. Harris found that stimulation of specific regions of the hypothalamus and anterior pituitary caused ovulation, from which he hypothesized that nerve fibers from the hypothalamus mediated the response to the hypophysis. Harris went on to invent a 2-electrode system capable of stimulating the hypothalamic brain region over several days and weeks remotely in conscious animals. His seminal invention used a skull-fixed insulated coil of enameled copper wire connected to an insulated platinum wire stimulating electrode that descended into the brain. A silver plate placed beneath the scalp and over the frontal bones served as a second electrode (23). The animal with the implanted coil was placed in a strongly fluctuating magnetic field to achieve electrode stimulation. Following years of technological refinement of the system and numerous collaborations, Harris elucidated the adenohypophysis's innervation and blood supply. Harris' work culminated in providing definitive evidence that a neurohormonal factor, traveling through the adenohypophysis portal system, was essential for controlling hormone production in the pituitary (24).

Emanating from Cannon's work, and during Harris's time, the endocrinologist Selye became known for conceptualizing the first stress model, known as General Adaptation Syndrome. Selye published widely on General Adaptation Syndrome, using his work and his peers (9) to support his proposal of a 'syndrome of diverse nocuous agents' (25). Selye describes three stages of responses to acute, non-specific damage he called “stressors” (26, 27), that ranged from excessive muscle use to spinal shock (25). The first phase, known as the alarm response, is what we would identify today as the “fight or flight” response coined by Cannon (28). The second phase is the stage of resistance, defined as a continued state of arousal, and the third phase is exhaustion. Selye eloquently articulated stress as “passive non-specific damage intricately mixed with those of active defense,” which gradually eroded health over time as the body's neurological and endocrinological defense systems continually responded to and became exhausted by stressors.

The foundation of neuroendocrinology provided by Harris' work was significantly advanced when a trainee of Selye, Guillemin, and Schally independently demonstrated that a hypothalamic factor whose chemical structure was not yet defined stimulated the release of adrenocorticotropin releasing hormone (ACTH) from the pituitary. Thus, emerged the first description of the corticotropin-releasing factor (CRF). The scientific lineage of Selye's work, from his trainee, Guillemin, and later, Guillemin's trainee, Wylie Vale, would define the adrenal-hypothalamic-pituitary response to stress as we know it today (29).

### LC-NE Anatomy and Physiology of the Stress Response

The initial conception in 1931 by Ernst Ruska, and the later application of the transmission electron microscope (TEM) to biological samples in the 1940s, enabled scientists to observe the subcellular structure of neurons for the first time (30). The use of thin tissue sectioning combined with plastic embedding of samples made it possible to keep organelles in their natural subcellular compartments (31, 32). These early ultrastructural studies provided unequivocal support for the neuron doctrine by capturing micrographs of individual neural cells (33). TEM studies identified synaptic contacts' morphological features and confirmed dendritic spines' existence; a feature previously observed using the Golgi Method (34). High-resolution TEM analysis of axon terminals led to the identification of two types of neurotransmitter -containing vesicles; small synaptic vesicles (SSV) later shown to store and release fast-acting transmitters such as glutamate and GABA, and large dense-core vesicle (LDCV), the primary site for neuropeptide storage and release (35). Monoamine transmitters, such as norepinephrine, can be stored in either SSV or LDCV (36). TEM studies also provided evidence for putative extrasynaptic release of LDCV (36, 37) and non-synaptic transmission or volume transmission (38). The theory of volume transmission was supported by anatomical studies of the time that indicated neuropeptide receptors were not frequently found at the synaptic cleft, thus raising the possibility of asynaptic peptide release, distant receptor binding, and activation of neurons or glial cells in the microenvironment (39). Additionally, the low synaptic incidence of monoaminergic terminals and their receptors' extrasynaptic localization (40–42) suggest that these transmitters may be preferentially released in a manner consistent with volume transmission.

The work of Coons et al., who first used fluorescein-labeled antibodies to localize antigens at the light microscopic level, inspired increasingly advanced antigen-detection methods (43, 44). The introduction of the radio immuno-assay for direct immuno-labeling of cellular components by Yalow and Bernson and the subsequent development of the immunocytochemistry indirect labeling techniques provided the tools necessary for cell-type-specific labeling of neurons by antibodies (45–47). Further, Singer's development of an antigen visualization technique that employed electron-dense substances such as colloidal gold conjugated to the antibody to improve its detection under the electron microscope significantly increased TEM studies' success (48). Under the electron microscope, the electron-dense colloidal gold particles are easily observed as dark puncta and quantified using the TEM (49). Another significant advancement in this regard was the discovery and subsequent widespread use of Horseradish peroxidase (HRP) as an enzymatic label for the detection of biomolecules in immunohistochemistry (50). The common use of HRP has been attributed to its high enzymatic activity, that when combined with electron-dense chromogen substrates such as 3,3α-diaminobenzidine tetrahydrochloride, created a substantial reaction product that could be easily visualized under a light or electron microscope (51).

A line of neuroanatomical studies employed indirect labeling techniques to visualize Tyrosine Hydroxylase (TH), the first and rate-limiting enzyme of catecholamine biosynthesis (52). These seminal studies approximated dopaminergic and noradrenergic neuronal cell bodies (53, 54). The LC is localized in the fourth ventricle base and is known for its vast and divergent efferent system, whose noradrenergic fibers reach nearly the entire neuraxis (53). NE is stored in both SSVs and LDCVs that can be localized throughout the somatodendritic processes LC neurons (55–57). These findings were preceded by the stereotaxic mapping of the ascending monoaminergic neurons (58) and followed with advanced immunohistochemical studies that employed high-resolution immunoelectron microscopy to visualize the cellular and subcellular distribution of TH within LC neurons (59, 60). Later studies compared the immunohistochemical labeling of TH and dopamine-β-hydroxylase (DβH), the final enzyme in NE biosynthesis, to classify catecholaminergic neurons into distinct populations of noradrenergic and dopaminergic neurons (61). Subsequent high-resolution electron microscopy analysis of the LC revealed CRF-immunoreactive axon terminals that formed synaptic specializations with rostral LC dendrites (62), and were later found to be primarily co-localized with excitatory amino acids (63), suggesting that CRF afferents to this region directly control LC neuronal excitability and activity (64).

Wylie Vale et al. seminal work characterized a 41-residue peptide found to induce corticotropin-like and endorphin-like immunoreactivity in the anterior pituitary cells. Subsequent studies by this group characterized CRF-immunoreactive nerve fibers in the hypothalamus (65), its effects on the sympathetic nervous system and metabolism (66), and its role in stimulating the release of ACTH during stress (67). Further, Vale's group identified the CRF receptor 1 (CRFR1) and three urocortin receptors (68). Vale's continued study of the system led to the discovery that human patients with major depression had elevated CRF (69) and that stress could inhibit reproductive function in the rat (70). Other fascinating studies found that CRF could stimulate the secretion of the chemokine IL-1 involved in the immune response under conditions of stress (71). In many ways, Vale et al. findings echo and further validate Cannon and Selye's observations of the profound effects of stress on the body.

At this time, scientists believed CRF could mediate stress-induced LC activation based on anatomical evidence that demonstrated dense CRF-immunoreactive fibers within the dendritic pericoerulear regions surround the LC core (72). The refinement of axonal tract-tracer technology enabled a complete understanding of this region and others, as it allowed for examining connectivity between brain regions. Tract-tracing experiments can employ two types of tract tracers. The first, anterograde tracers, are taken up into the cell via endocytosis in the somatodendritic processes of neurons and then transported to their axon terminals where they may be detected using various immunohistochemical techniques including TEM. Alternatively, the second type of tract tracer travels retrogradely, as axon terminals endocytose the tracer and transport it back to the soma. The first anterograde tract tracer (73) was derived from the kidney bean lectin Phaseolus vulgaris leucoagglutinin (74) in 1978. Retrograde tracers, including fluorogold and wheat germ agglutinin, are commonly used retrograde transporters that have been used in combination with TEM (75–80). More recent approaches to mapping neural circuitry also include genetic tracers embedded within recombinant viruses that can travel across synapses to define multiple synaptic connections (81–83). Studies that employed tract-tracing techniques with immunocytochemistry revealed that dense bundles of neuronal fibers extend from diverse brain regions (84) and are composed of distinct neurochemical profiles that vary by brain region (85, 86).

Later physiological studies supported earlier neuroanatomical studies by confirming that CRF engages the LC during acute and chronic cognitive and physical stressors (64, 87, 88). These methods helped establish that the axon terminals of several brain structures release CRF onto the LC (72, 89). When presented with a stressful stimulus, afferents from the paraventricular nucleus of the hypothalamus release CRF onto the anterior pituitary (90) and onto LC neuronal cell bodies and dendritic zones (91), regions of the LC densely populated with CRFR1 (92). The activation of this pathway during the stress response came to be understood as a parallel but intricately connected process that occurs alongside the HPA axis-mediated peripheral stress response. Depolarization of LC neurons results in the production and release of NE from axon terminals throughout the neuraxis (93, 94). This has been demonstrated in microdialysis studies investigating the effects of restraint, tail shock, auditory, and hypotensive stressors on extracellular levels of NE in the terminal areas of the LC (95, 96). Of particular interest are the afferents expressing CRF from the central nucleus of the amygdala, which are thought to activate the LC to engage cognitive processes in response to environmental stressors (72, 91) thus, has been conceptualized as the cognitive limb of the stress response (64).

### LC-NE Anatomy, Physiology, and Function in Arousal

Initially described in 1929, Berger described an instrument capable of measuring electrical activity waves that send pulses across the brain (97). Today, those electrical pulses are known as neuronal oscillations, and they are measured as a frequency in Hertz (Hz). Because the EEG directly measures neural activity with very high time resolution, it is considered an outstanding tool for studying the broad range of neurocognitive processes that dictate human behavior (98). When the full potential of the EEG came to fruition, it brought revolutionary advancements to the scientific research community as investigators across disciplines characterized the stages of the sleep-wake cycle, epileptic seizures, and for the first time, glimpsed global brain dynamics.

The EEG provides information about the speed of an oscillation (frequency), the amount of energy in a frequency band (Power, expressed as amplitude^2^), and the amount of synchronization across neurons (Phase; measured in radians). These are the essential elements of modern neural dynamics and set the foundation for the rapidly growing field of Neural Field Theory. Five EEG frequency bands are defined by the frequency ranges in which they occur and are associated with diverse cognitive processes. The delta band (1–4 Hz) is the slowest but has the highest amplitude and is related to non-REM slow-wave sleep (SWS) (99). The theta band (4–8 Hz) is associated with mental tasks that require a high degree of focus, such as learning or recalling information (100). Meanwhile, the alpha-band (8–12 Hz) that was first described in Hans Berger's initial 1929 publication is now associated with sensory, motor, and memory functions and is known to be active during states of mental or physical relaxation while the eyes are closed (100). In contrast, with open-eyes and in a task-focused state, alpha frequency waves are suppressed. The higher frequency Beta Band (12–25 Hz) is associated with anxious thinking or active concentration (101), and while less is known about the highest frequency gamma-band (>25 Hz) (99), abnormalities in this frequency are common in schizophrenia and likely reflect large scale network dysfunction (102).

The use of the EEG in research observing brain states provided the first evidence for the involvement of the LC and other brainstem structures in arousal. The pivotal work of Moruzzi and Magoun revealed that stimulation of the reticular formation elicited an excitation that ascended from the lower bulbar region of the brainstem, through the pons, midbrain tegmentum, and into the caudal diencephalon (103). Moreover, the activation of these brain structures resulted in the switching of EEG high-voltage slow-wave activity to low voltage fast activity. Importantly, the resulting ascending activation resulted in the de-synchronization of cortical structures. As clinician-scientists, Moruzzi and Magoun were struck by the similarity of this response to the subtle but significant alpha-wave blockade of EEG activity that was commonly observed when patients turned their attention to a visual stimulus and characterized the state transition from sleep to wakefulness (103). The thalamic and reticular regions involved in this activation response collectively became known as the reticular activating system (RAS). The degree of RAS activation could induce three distinct arousal states: waking, SWS, and rapid eye movement (REM) sleep (104).

Subsequent lesion studies implicated a mid-pontine, pre-trigeminal area of the brainstem in behavioral state-dependent EEG activity that was varied when the animal was awake and alert but synchronized during sleep (105). Subsequent pharmacological studies indicated that arousal EEG patterns induced by injection of NE had an activation site at the midbrain level (106), showing a close relationship between thalamic and reticular sites during sleep-wake cycles (107). Other physiological properties of LC neurons came to be understood by electrophysiological studies enabled by the development of tungsten electrodes. Hubel's advancement to probes composed of sharpened tungsten enabled physiologists to record much smaller neurons and axons (20, 108). Now armed with pharmacological agents, EEG, and electrophysiology, neurobiologists had the unprecedented insight into the brain's neural underpinnings, which allowed for a closer, more detailed understanding of small groups of neurons such as the LC. In a seminal study of this nature, Chu and Bloom used microelectrodes combined with EEG to correlate LC discharge activity with global brain wave activity in parallel with behavioral observation and reported sleep-stage dependent changes in discharge patterns (109). Later studies by Aston-Jones and Bloom not only confirmed but also more accurately described the involvement of LC-NE activity in RAS. Their findings demonstrated that LC tonic discharge rates were highest in the waking state, slower in SWS, and absent in REM sleep (110), thus firmly establishing a foundation for LC-NE involvement in the RAS that controls sleep-wake cycles.

It is important to note that the spectrum of arousal states is the result of a distinct balance of multiple transmitters derived from several brain structures. The discovery of Orexin peptides in the lateral hypothalamus (111) and the subsequent immunohistochemical characterization of their distribution throughout the CNS were the first to suggest a complex multi-transmitter integrative system controlled arousal (112). Dense orexin-immunoreactive fibers in the LC were found to have a significant excitatory effect *in vitro* and were demonstrated to have an essential role in mediating transitions of sleep to wake *in vivo* (112–114). Orexin also innervates the basal forebrain, a primarily of cholinergic and GABA-ergic nucleus that is also sufficient for cortical activation and transition from NREM sleep arousal. Although orexin has different effects on cholinergic and non-cholinergic cells of the basal forebrain, both stimulate the transmission of acetylcholine in the cortex, resulting in stimulating wakefulness [reviewed in (115)]. Interestingly, while orexin clearly plays a pivotal role in wakefulness and arousal, it is considered a stabilizer of wakefulness rather than a sole determinant. This stems from selective optogenetic studies that demonstrate an inability of orexin to stimulate sleep to wake transitions under conditions of selective LC silencing. Thus, it has been proposed that a common theme of orexinergic actions is the integration of homeostatic or external goal-oriented signals of danger or reward to promote motivation (116).

Early studies on LC stimulus-evoked neuronal responses in unanesthetized monkeys indicated that iontophoretically applied NE was more effective in reducing spontaneous cortical activity “noise,” to a greater extent than it could reduce the activity evoked in response to the stimulus “signal” (117). Moreover, during excitatory responses, NE reduced a more significant proportion of low-discharge rates than high-discharge rates in cortical regions (117). This was a critical discovery, as it suggested that NE could silence some signals while enhancing others. Further physiological studies in the awake rat demonstrated that LC-NE neuron activity varied as a function of sensory stimulation and arousal. These studies were the first to show that LC neurons responded vigorously to mild, non-noxious, physiologically relevant stimuli, and the previously established responses to noxious stimuli under anesthesia (118). Notably, electrical stimulation increased neuronal responses to strong or preferred stimuli while decreasing responses to weak inputs, thereby enhancing “signal-to-noise” ratios in target cell impulse activity (110). This concept pervades the LC-NE literature even today, as interpretations evolve with the field (6, 119).

A critical line of inquiry still under investigation is in determining how salience is encoded and how a system the system involved in such encoding filters and initiates memory processing in the hippocampus. Previous studies had demonstrated that phasic activation of the LC produced a protein synthesis-dependent long-term potentiation 24 h after stimulation, suggesting that phasic firing of the LC could encode the initiation of long-term memory processes (120). Subsequent studies discovered a mechanism by which the LC may facilitate such an effect by identifying two sub-populations of functionally distinct inhibitory interneurons in the dentate gyrus that respond to NE in an opposing manner (121). It was demonstrated that NE could increase the excitability of one population of interneurons while decreasing the excitability of the second population of interneurons (121).

### The Evolution of Arousal Theory

Cognitive psychologists focused on measures of attention and arousal in their study of “stress,” resulting in the theoretical emergence of a two-stage process of stimulus-related information processing required for a stress response to occur. The first stage, termed the “evaluative reflex” (122), was believed to be an automated, non-specific response to environmental stimuli followed by the second stage of higher-order cognitive information processing. Here, many studies utilized experimental paradigms that assessed the impact of various levels of increasing task difficulty or mental load during tests of visual attention. Early studies suggested that an optimal level of stress, embodied by the inverted-U shape curve, could be achieved to maximize performance on cognitive and physical tasks (123). While this original study has been heavily scrutinized in more recent years (2, 123), ultimately, multiple lines of evidence converged on the idea that moderation was key. Easterbrook's studies of human performance in the context of stress supported the notion in his observations of his personal experiences that suggested, while long-term (chronic) stress was detrimental, a certain amount of short-term (acute) stress could be beneficial in performing physical and cognitive tasks (124). Stress neurobiologists of today have generally come to accept a conceptual paradigm in which stress and workload can elicit a reduction in an individual's perceptive field or ability to scan the environment for cues, thus creating a “tunnel” of attention.

One of the most significant concepts of this time, the Tunnel Hypothesis, was guided by Easterbrook's observations and others in the study of attention and information processing under conditions of stress. In line with this rationale, the continued study of arousal and the processing of auditory stimuli brought to light an understanding that noise could increase arousal and result in a narrowed attention span (125). In other words, in the aroused state, information processing becomes limited to stimuli related to environmental cues, particularly those that may signal threat, such as noise. This led to the first assertion that arousal may moderate information processing during stress (2). The work of Beatty supported this notion, demonstrating correlated changes in pupil diameter with increases in task difficulty, finding that pupil dilations had a significant relationship to information processing (126). From this direct relationship, these investigators inferred that pupil dilation could be an indicator of resource mobilization. To further understand how stress may hinder performance, Rachman et al. studied performance on cognitive tasks under conditions of bomb disposal. Subjects were described as experiencing significant physiological changes consistent with anxiety and fear, including increased heartbeat, labored breathing, and trembling (127). The subsequent observation that once a stress or anxiety response was provoked, mental resource allocation could be diverted away from the task at hand and toward task-irrelevant stimuli, resulting in performance deficits (128). This idea is still relevant today as cognitive psychologists continue to discuss theoretical mechanisms for selective attention and the ability of stress to both improve or weaken performance in a state-specific manner.

In his theory of “stress,” Lazarus influenced the field by suggesting that psychological stress occurs when a situation is perceived as threatening (129). This concept gained importance over time, as Kahneman put forth the notion that cognitive processing required mental resources that are limited in quantity (130), and was coined by Norman and Bobrow as the limited-capacity resource model (131). Thus, Kahneman conceptualized cognitive tasks as processes that compete for resources that are allocated based on the energetic requirements for the neuronal populations involved (metabolism of glycoproteins or blood flow/oxygenation) (132, 133). Ultimately, this came to be understood from an evolutionary perspective that echoed Lazarus's focus on “threatening” stimuli. It was conceived that cognitive tasks required for- or related to- survival would be prioritized in terms of importance and resource allocation. This ideology likely influenced the concept of “salience,” which describes the prioritization of a stimulus or mental representation based on its importance in achieving a goal. In the case of a threatening stimulus, survival-related actions or stimuli would be designated as a high priority. The related “neural representations” would be conveyed as salient information, and therefore would be granted more resources to survive. Wickens et al. asserted that the performance of tasks was fundamentally dependent on a pool of limited resources, using capacity, attention, and effort as examples (134). Wickens went on to create a formula for optimal performance, in which he proposed that optimal performance would be equal to the resources available divided by the task difficulty (135).

The late 90's into the early 2000s brought some clarity in the conceptual delineation of arousal from stress. Razmjou provided a framework for the concept of arousal (136), asserting that, “arousal is a hypothetical construct that represents the level of central nervous system activity along a behavioral continuum ranging from sleep to alertness” (p. 530). This concept was further developed with a comprehensive definition of stress, in which Wofford and Daly proposed that there were three domains of a stress response. The first domain, physiological arousal, included physiological indices such as heart rate, blood pressure, and temperature. The second domain composed psychological responses such as dissatisfaction, anxiety, sleep problems, depression, or irritation (137). Finally, the third domain was that of behavioral responses, for example, job performance, drug abuse, eating disorders, aggression, or poor relationships (2, 137). Moreover, Gaillard et al. used the concept of energy mobilization to distinguish the arousal further- related mental load from stress, stating that mental load represents the energy mobilization required to adapt to an environment in a healthy manner, while stress induces a heightened state of activation that, presumably requires increased energy mobilization, and fails to improve performance (138). Thus, while the field of Cognitive Psychology did not determine a unitary model of stress, the work of many researchers converged on the multifaceted nature of arousal, that, when paired with a perceived inability or insufficient resources, could manifest as a stress response that was composed of physical, cognitive, behavioral or performance characteristics.

Later, a growing interest in the effects of stress on attention led to the concept of active and passive attentional states that were driven by top-down and bottom-up information processes, respectively. Ohman et al. asserted that top-down processing implies a voluntary action in which the organism directs attention, while bottom-up processing was a stimulus-driven process that involved environmental cues to draw-in attention (139). The renewed interest called back to earlier models of attention that focused on a reflexive evaluation followed by an appraisal by higher-order cognitive processing. Rohrbaugh viewed the purpose of the orienting reflex as preparation for stimulus perception (140), and it likely occurs before higher-order cognitive assessment (141). Additionally, Sokolov and Vinogradova suggested that once a given stimulus is detected, it enters into a pattern recognition system through which it is compared to a pre-existing “library of internal representations (neuronal models) of previous stimulations” [(133), p. 324]. Further developments from Crawford and Cacioppo examined the asymmetrical and negative bias that humans have toward the automatic processing of information, concluding that humans are wired to evaluate the environment and that this evaluation likely takes place subcortically, before any conscious awareness of emotion or higher-order cognition occurring (122). The importance of these developments will be clarified in future sections that describe the role of the LC-NE system in information processing.

### The LC-NE System and Allostasis

The LC-NE system is uniquely positioned to influence global brain states, evidenced, at least in part, by the finding that NE can be released from synaptic boutons to effectively diffuse beyond the bounds of a typical synapse to interact with adrenergic receptors (AR) on surrounding neurons and glial cells (142, 143). This becomes particularly important when considering the protective role of NE in modulating central inflammatory responses (144). Thus, the design of LC neural architecture reflects its broad functionality, as it is critical for promoting attention, wakefulness, and cognition (145), processes that require the coordination of multiple brain regions across large scale networks (146, 147). Several lines of evidence indicate that LC activation is sufficient to initiate and maintain states of arousal, and LC activity is positively correlated with vigilance (148) and attentiveness, supporting the idea that the LC mediates scanning of the environment for potentially threatening stimuli (64, 145). Further, LC discharge frequency indicates state changes in arousal and attention processing. For example, low rates of tonic activity suggest a state of potential hypo-arousal and is associated with disengagement from the environment (145). In contrast, optimal tonic activity levels reflect an arousal state associated with high responsivity to sensory stimuli in the environment. Optimal tonic activity in this state enables phasic burst firing, in which electrotonically coupled LC neurons fire together in synchrony (149, 150). This level of arousal is associated with focused attention on task-related, novel, or unpredictable stimuli (145, 146, 151).

Amidst the progress of charting the involvement of CRF in stimulating the LC-NE system in the physiological response to stress, Sterling and Schulkin and Sterling introduced the concept of allostasis as a process of reestablishing stability in response to a challenge (152, 153). Shortly after, Bruce McEwen built upon this concept by introducing the concept of “allostatic load” and “allostatic overload” (154). McEwen argued for the reinterpretation of Selye's General Adaptation Syndrome based on the understanding that stress could have protective and deleterious effects on the body. McEwen reinterprets Selye's alarm response as “the process leading to adaptation, or allostasis, in which glucocorticoids and epinephrine, as well as other mediators, promote adaptation to the stressor.” Perhaps the most significant reinterpretation arises in McEwen's view of the third stage, in which he asserts that exhaustion of defense mechanisms was insufficient to account for the alterations in the body. Instead he proposed that at a certain point, the stress mediators themselves no longer served a protective role, but rather became counterproductive, exacerbating damage to the body. Thus, noting that the word “stress” is overused and imprecise, he introduced the term “allostatic overload,” defined as circumstances under which the hormones of the HPA axis, catecholamines, cytokines, and other physiologic mediators are over-worked, resulting in active contributions to the cumulative effects of daily life. Importantly, McEwen's framework emphasized the wear and tear of the regulatory systems caused by these mediators in the brain and body (155). McEwen states further that allostatic overload is a state in which physiological mediators are no longer purposeful, and these circumstances predispose individuals to disease (156, 157).

## Modern Integrated Perspectives of Arousal and the LC-NE System

### The Growing Field of Large-Scale Network Dynamics

The emergence of brain imaging technologies, first Positron Emission Topography (PET) in 1974, and subsequently Blood Oxygen Level Dependent (BOLD) functional magnetic resonance imaging (fMRI) in 1990, enabled scientists across the spectrum of neurobehavioral research to collect and analyze precise spatial information about the brain regions that gave rise to cognitive processes [(158); reviewed in (159)]. From these developments, emerged a number of new strategies to process and interpret fMRI data (160). Seed-based correlation analyses deliver information about the activity of a pre-selected region of interest in relation to the rest of the brain. Principal Component Analysis is another common univariate approach that focuses on the high-resolution spatial component of the data rather than the limited temporal information available (161). These analyses were sufficient to enable researchers to couple a specific observed cognitive task or deficit to a localized brain region. However, because BOLD is a fundamentally metabolic measure of neural activity, the temporal specificity of these scientific observations is limited by hemodynamic response time. This translates to the identification of broad stimulus-response areas of the brain without an understanding of the functional specificity of each brain region that could only be parsed out with high temporal resolution.

A pivotal advancement came with the discovery that fMRI and EEG could be used in combination to derive high-resolution spatio-temporal information about neuronal events at the timescale of tens of milliseconds (162). This advancement necessitated the development of more complex analytical systems that are capable of collecting, processing and integrating large data sets. The advancement of computational neuroscience has addressed this need, as model-based (163) and data-driven EEG-fMRI fusion techniques, including independent component analysis (ICA) and canonical correlation analysis, have been described (164, 165). Structure-function connectome analyses of EEG-fMRI data using graph theory translated to maps of nodes (brain regions) connected by edges (network connections). The strength of within-network connections (between nodes in the same network) could be represented by assigning a weight based on the correlation coefficient between the two brain regions (160). This enabled researchers to hypothesize about the time-dependent functional roles of specialized regions within a system that can dictate complex behavioral responses when brought together at a network level. This conceptual line of inquiry, combined with continued methodological advancements, shifted our understanding of the neural basis for cognition. The advent of ICA moved the field away from univariate models that coupled specific deficits to individual brain regions and toward multi-variate approaches. ICA uses global EEG-fMRI temporal and spatial data to identify Intrinsic Connectivity Networks (ICNs), sub-components of the data composed of highly connected large-scale brain regions whose activity could be reliably observed during a specific set of cognitive responses (166).

#### Identification and Characterization of Intrinsic Connectivity Networks

**Figure 1:**
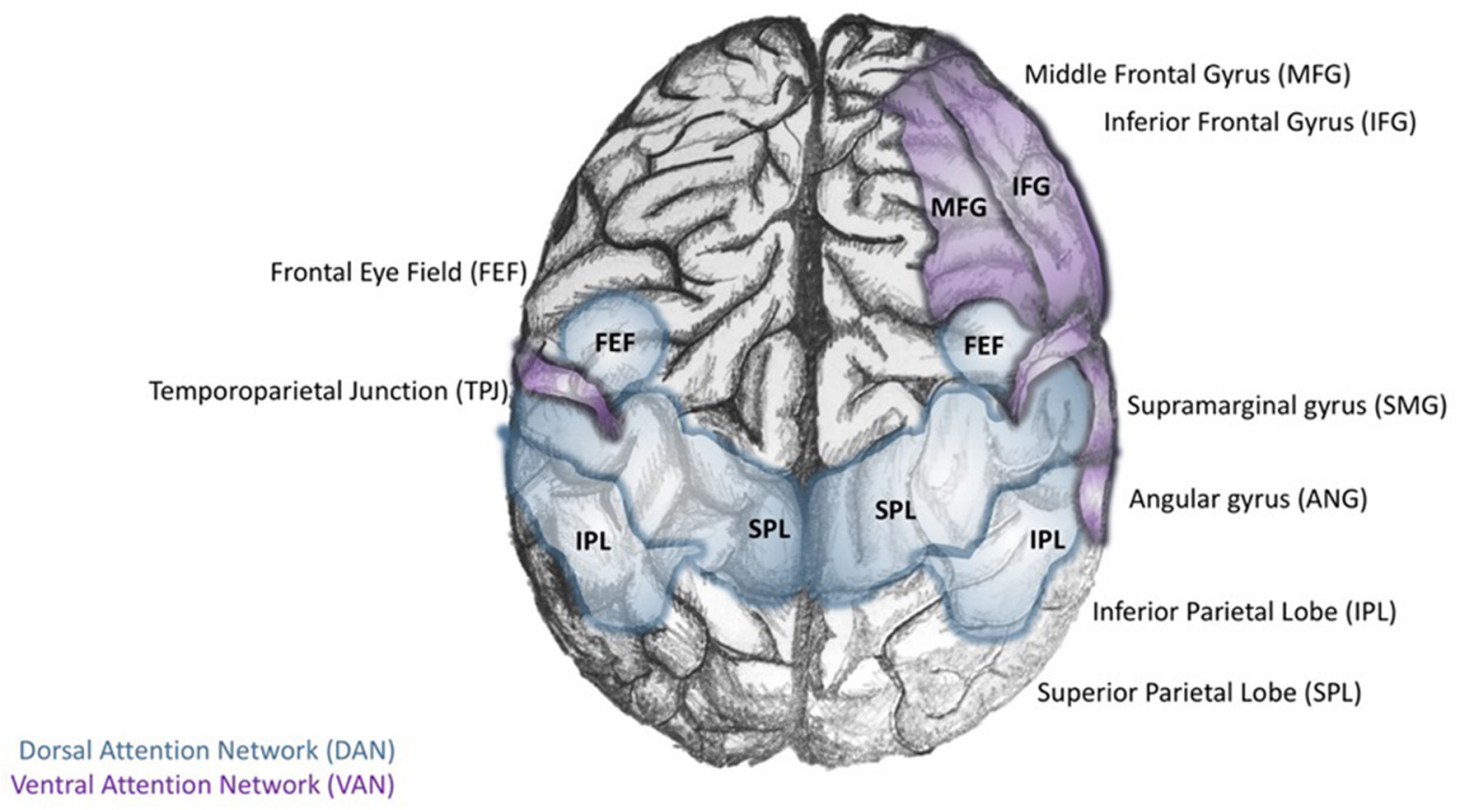
Core regions of the dorsal and ventral attention networks. The Dorsal Attention Network (DAN; blue) is defined by the interconnectivity of the dorsal posterior parietal lobe (dPPL) and dorsal frontal cortex [dPFC-Frontal Eye Field (FEF)]. The activity of the left dPPL may be a preparatory step for stimulus-response tasks; selective attention to task-relevant stimuli results in the development of cognitive cues. Meanwhile, activity in the (dPFC-FEF) coordinates motor responses to the task-relevant stimulus. In contrast, the Ventral Attention Network (VAN) is anchored in the right temporoparietal lobe (TPL), and ventral frontal cortex (vPFC; composed of the inferior and middle frontal gyri and the frontal operculum).

In their synthesis of Arousal Theory with PET and BOLD fMRI data available at the time, Corbetta and Shulman build on the concept of top-down and bottom-up processing of information during the orienting reflex, suggesting that attention is controlled by two neuroanatomically defined systems (167). The Dorsal Attention Network (DAN) relies on top-down cognitive information processing to build task-related stimulus-response maps that pair cognitive cues with associated motor responses. In contrast, the Ventral Attention Network (VAN) is concerned with the salience or novelty of a stimulus in the environment (168). In this system, bottom-up sensory cues in the environment can interrupt an ongoing task-related cognitive activity to quickly reorient attention (within 50 ms) to novel or infrequent events. In broad strokes, Corbetta and Shulman argue that DAN is primarily an “endogenous orienting system” while VAN is an “exogenous orienting system” (Figure 1). Considering the trajectory of this line of research, the authors provide a functional interpretation of data beyond their time. This is relevant in light of the findings of Schupp et al., who affirmed the earlier ideas of cognitive psychologists that conceptualized a two-stage process of stimulus-related information processing, including an automatic “evaluative reflex,” followed by a stage of higher-order cognitive information processing (169). While investigating electrocortical activity during the processing of emotional images using event-related potentials and fMRI, these investigators found that the processing of emotional images is related to the degree to which the stimuli emotionally engage individuals. Those with strong affective cues were processed more for both pleasant and unpleasant images. It was determined that selective attention to the stimulus's location occurred within 100 ms and that attention to its features such as color, orientation, and shape occurred between 150 and 200 ms (169).

The continued investigation of cognitive processes using combined fMRI and EEG led to the discovery of the first resting-state large-scale brain network. Studies in non-human primates and humans converged to identify the Default Mode Network (DMN), defined by core regions that were reliably activated during internally focused tasks such as forming perceptions of others or retrieving memories (170–172). The discovery of the DMN brought about a new line of investigation concerning the maintenance and switching between brain states, particularly transitions from DMN-mediated internally focused activities to those that involved the external environment (173). In this regard, Corbetta and Shulman's DAN could account for attentional control involved in the preparation, coordination, and execution of motor responses to task-related stimuli, while the VAN could facilitate task switching upon exposure to a novel or infrequent sensory stimulus in the environment (167). During this time, an ICN engaged selectively during working memory, problem-solving, and decision making, was discovered and called the Central Executive Network (CEN) (174).

**Figure 2:**
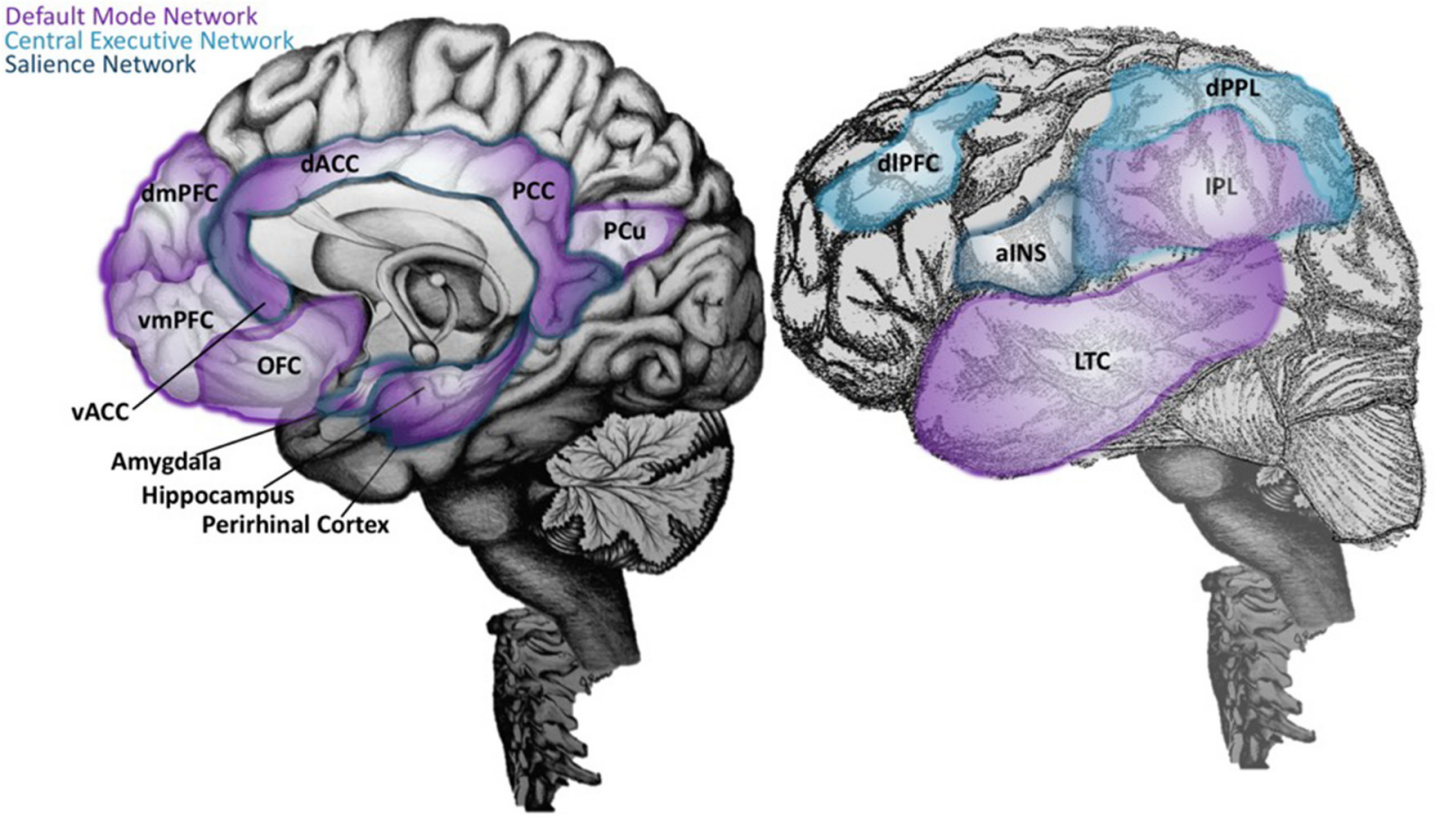
Neuroanatomical structures of the Default Mode, Central Executive, and Salience Networks. On the right, an illustration of the brain with mid-sagittal view reveals key neuroanatomical structures of the Default Mode Network (DMN) (purple), which includes the ventral medial prefrontal cortex (vMPFC; or infralimbic region), posterior cingulate cortex (PCC), precuneus (pCu), inferior parietal lobe (IPL), lateral temporal cortex (LTC), the dorsal medial prefrontal cortex (dMPFC), and the hippocampal formation (170, 171). The most recently evolved dorsolateral prefrontal cortex (dlPFC) and the lateral posterior parietal lobe (PPL) are key regions of the Central Executive Network (CEN; light blue) (174), and are depicted in the lateral view of the brain in the left panel. In the same panel, the anterior insula (aINS) is labeled, along with other regions of the Salience Network (SN; dark blue) that are visualized on the mid-sagittal illustration in the right panel. This includes the dorsal anterior cingulate cortex (dACC), amygdala, and ventral tegmental areas. The SN detects, integrates, and filters incoming sensory, autonomic, and emotional information to determine the relative importance of a stimulus (4, 176). Regions labeled with two or more colors are overlapping network areas that are believed to be important for between-network interactions.

Regions of the VAN are often combined with several limbic structures to collectively form the Salience Network (SN), which detects, integrates, and filters incoming sensory, autonomic, and emotional information to determine the relative importance of a stimulus (4, 8). These discoveries shifted our understanding of the neural basis for cognition to a paradigm focused on a balance between three core ICNs: the DMN, CEN, and SN (8). In this triple network model, each ICN serves a specialized function, that when optimally coordinated, results in the emergence of cognition, goal-directed, and stimulus-directed behavior [(8, 175); Figure 2].

#### The Hierarchy of Large-Scale Networks Dictates their Functional Balance

In terms of network analysis, the strength and direction of a relationship between large scale networks are represented as edges weighted by between-network correlation coefficients. Importantly, in addition to positive correlations (excitatory/engaged), there can also be negative or anti-correlation (inhibitory/disengaged) between edges (177). Anti-correlations were once thought to be an artifact; thus, many forms of data “optimization” methods only took positive values into account by pre-processing the data using binary thresholds in which values below the threshold became zero and above the threshold became one (178, 179). However, several recently developed approaches, such as Path Length Associated Community Estimation [PLACE; (180, 181)], and Probability Associated Community Estimation [PACE; (177)], that do not use thresholds or signal-magnitude for correlations overcome these challenges. In particular, PACE is designed to interpret higher probabilities of an edge being correlated or anti-correlated as optimal relationships to capture because regardless of their direction, those relationships are more likely to reflect connectivity between different regions or different networks (177).

These factors become important when considering how large-scale networks interact with each other. Based on their specialized functions, it is advantageous for large-scale networks to operate independently, in synchrony, or in opposition, under various circumstances. The DMN, known to be engaged during stimulus-independent tasks or those related to internal thought, is usually suppressed during CEN activation (182–184). While mechanisms of large-scale network suppression are not well-understood, it is well-established that the suppression of opposing networks such as the DMN and CEN is crucial for the function of specific cognitive processes, including focused attention, working memory, and other executive functions (185). In line with this, a reduced ability to suppress DMN activity or effectively switch between DMN and CEN states has been linked to a wide range of cognitive and psychiatric disorders (8, 170).

A recent study employed hierarchal cluster analysis and spectral dynamic causal modeling to demonstrate that core regions of the SN and DAN that terminate in core regions of the DMN were negative (inhibitory), while connections arising in the core of DMN that terminate in SN or DAN regions were weakly positive (excitatory) (186). Moreover, there were positive (excitatory) bidirectional connections between SN and DAN. Further analysis of the effective connectivity matrix confirmed that the SN was highest in the hierarchy, suggesting that it may play a role in switching between anti-correlated networks (186). It is important to note, however, that these authors included the left and right anterior PFC, including the dlPFC (BA9), which is typically considered a core hub of the DMN. Indeed, the large-scale network literature has inconsistencies in nomenclature, as well as diverse analytic methods, that make direct comparisons between studies difficult (187).

### Is the LC-NE System a Master Switch?

In discussing their physiological study on LC responses under different stages of arousal in 1981, Aston-Jones and Bloom proposed that the LC-NE “system may serve to facilitate transitions between global behavioral states” (110). At the time, these investigators reported the spontaneous discharge of LC-NE neurons across the sleep-wake cycle. However, nearly 40 years later, the notion that the LC plays a role in transitioning between global behavioral states is a continued investigation.

Dalley et al. proposed that an influx of NE to the PFC for novel stimuli may signal a mismatch between action and reward to promote behavioral modification (188). An action of this sort would require a switch from the current activity to a new behavioral response, an observation that Corbetta and Shulman likened to the “circuit-breaker” function of VAN when unexpected or novel stimuli are detected [reviewed in (167)]. Further inspired by the work of Corbetta and Shulman, Aston-Jones et al., Pardo et al., and Morrison and Foote, suggest that the activity of the right-lateralized VAN depends on LC-derived cortical NE, which is more densely concentrated in the right hemisphere, and implicated in arousal, vigilance, and selective attention (167, 189–191).

In a severe departure from the heightening complexity of LC-NE functional theories at the time, Bouret and Sara put forth the elegant notion that the evolved LC-NE system operates analogously to a small number of synchronized neuromodulatory cells observed in crustacean (146). The crustacean neuromodulatory system can abruptly re-orient widespread neural networks to adapt to environmental conditions, a fascinating parallel to the widespread environmentally engaged activity of the LC system. Further, the two systems share global reaching targets capable of inducing abrupt, widespread changes in activity on a global scale (146). Together with an understanding of LC-NE system dynamics, investigators in several groups suggested that the LC initiates brain state changes in response to the environment to facilitate behavioral responses tailored to the most critical information. Thus, it has been proposed that the activation of the LC and release of NE terminates the resting state and begins a brain-state adjustment that involves cortical, subcortical, and autonomic activity, to facilitate focused attention (171, 173, 184, 192) and allows for behavioral output toward a task-related stimulus (119). Bouret and Sara propose a model in which the NE signal has a general reset function that mediates changes in widespread forebrain networks that are mediating specific cognitive functions. These investigators go on to present evidence using a multi-electrode dual recording of LC and cortical neurons to demonstrate that both within-trial and between trials, LC neuron depolarization occurs before forebrain neuronal activity and is closely related to shifts in cognition. Furthermore, the investigators demonstrate that LC-mediated stimulus-induced cognitive shifts do not occur in the absence of an external cue or when the presentation of a stimulus is predictable, thus delineating LC-NE induced changes are not a reflection of decision making or reward anticipation, but rather to facilitate the dynamic reorganization of neural networks to adapt to a changing environment quickly (146).

As previously discussed, during task states that do not require focused attention, the LC-NE system is characterized by a high tonic baseline, and investigators have proposed that this state is linked with the generation of spontaneous thoughts (193). In contrast, moderate LC tonic activity levels may promote optimal engagement in the environment to enhance performance in task- goal- or survival-oriented behaviors (119). Thus, it has been suggested that the deactivation of certain regions overlapping with DMN may be caused, at least in part, by high tonic activity associated with the LC-NE system, reflected by theta oscillations (194). A recent study utilizing a chemo-connectomics approach that employed Chemogenetic designer receptors exclusively activated by designer drugs (DREADDs), paired with resting-state fMRI, supports this notion (195). The investigators demonstrate that selective LC activation rapidly increases brain-wide functional connectivity in a manner consistent with AR distribution and the processing of salient information (195).

The opposing view does not disqualify LC-NE involvement in the switch *per se* but instead asserts that the anterior insula, a critical component of the SN, initiates the switch in behavioral states through large-scale network dynamics. This side argues that effective switching between brain states is at least partially dependent on the function of the SN, consisting of anterior insular and dorsal anterior cingulate regions (196, 197). In this light, a recent study examining the directional influences exerted by specific nodes of the SN on other brain regions concluded that the anterior insula plays a causal role in switching between the CEN and DMN, two networks that undergo competitive interactions across task paradigms and stimulus modalities and are thought to mediate attention to the external and internal worlds, respectively (196). Specifically, it was found that a touch stimulus resulted in activation of the mid-to-posterior insula, whereas anticipation of the touch stimulus activated the anterior insula and is correlated with the amount of activation in the caudate and posterior insula during information processing of the stimulus (198).

### Current Theories on Attention and Arousal

Few authors have attempted to explain the range of network, circuit, cellular, and molecular mechanisms involved in Emotion-Cognition human behavior theories. The two groups that made progress in this ambitious endeavor start by looking at human studies of the visual attention system and go on to describe parallel mechanisms for the selection of high priority stimuli. Both groups refer to a vast literature that, in addition to behavior, covers large-scale networks, local circuitry, neuronal populations, and single neuron responses to stimuli. Interestingly, the conclusions of both studies echo the findings of Aston-Jones and Cohen, who have long described “increased signal to noise” and “gain” as a characteristic of the LC-NE system (119).

The first study is primarily shaped around the concept of attention, which authors Buschman and Kastner define as the “selective prioritization of the neural representations that are most relevant to one's current behavioral goal.” These authors propose that this cognitive process arises from an “attentional modulation” system that interacts with pyramidal neurons and inhibitory interneurons to suppress the sensitivity of some stimuli while also increasing high-frequency synchronous oscillations associated with other stimuli. The authors further suggest that this mechanism primarily relies on lateral inhibition (5). While NE is not discussed in the review, the net effect of the proposed mechanism “results in increased sensitivity and decreased noise correlations.” The authors conclude that the cognitive process of attention is a result of increasing the inhibitory gain of the activated network (5).

**Figure 3:**
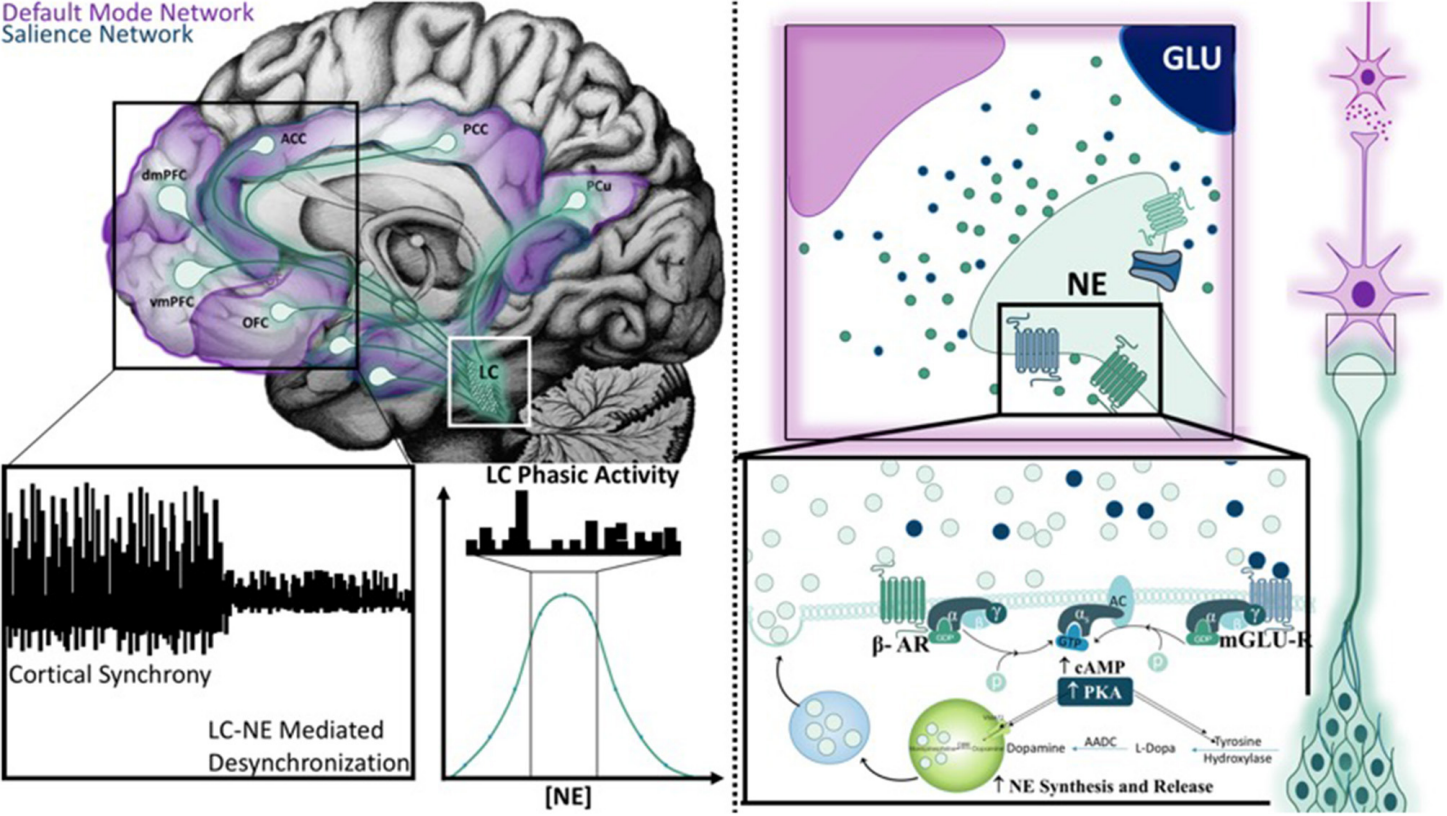
Mechanisms of the Glutamate Amplifies Noradrenergic Effects Theory Hot Spot. The mid-sagittal illustration in the top left corner is overlaid with relevant projection sites of the locus coeruleus system in the context of the default mode and salience networks. Novel stimuli or unexpected events results in a brief cortical desynchronization (as illustrated on the left bottom panel), and is thought to arise from phasic burst firing of the LC (middle bottom panel). The Glutamate Amplifies Noradrenergic Effects Theory asserts that glutamate and norepinephrine work together at “hot spots” to expedite signals from neuronal ensembles that represent salient information. Hot spots develop only when multiple action potentials release high levels of norepinephrine and glutamate is released from nearby terminals. Both signals are mutually enhanced through the activation of β-adrenergic receptors (β-AR), metabotropic glutamate receptors (mGLU-R), and ionic NMDA receptors. As a result, additional norepinephrine and glutamate is synthesized and released to create a hot spot.

**Figure 4:**
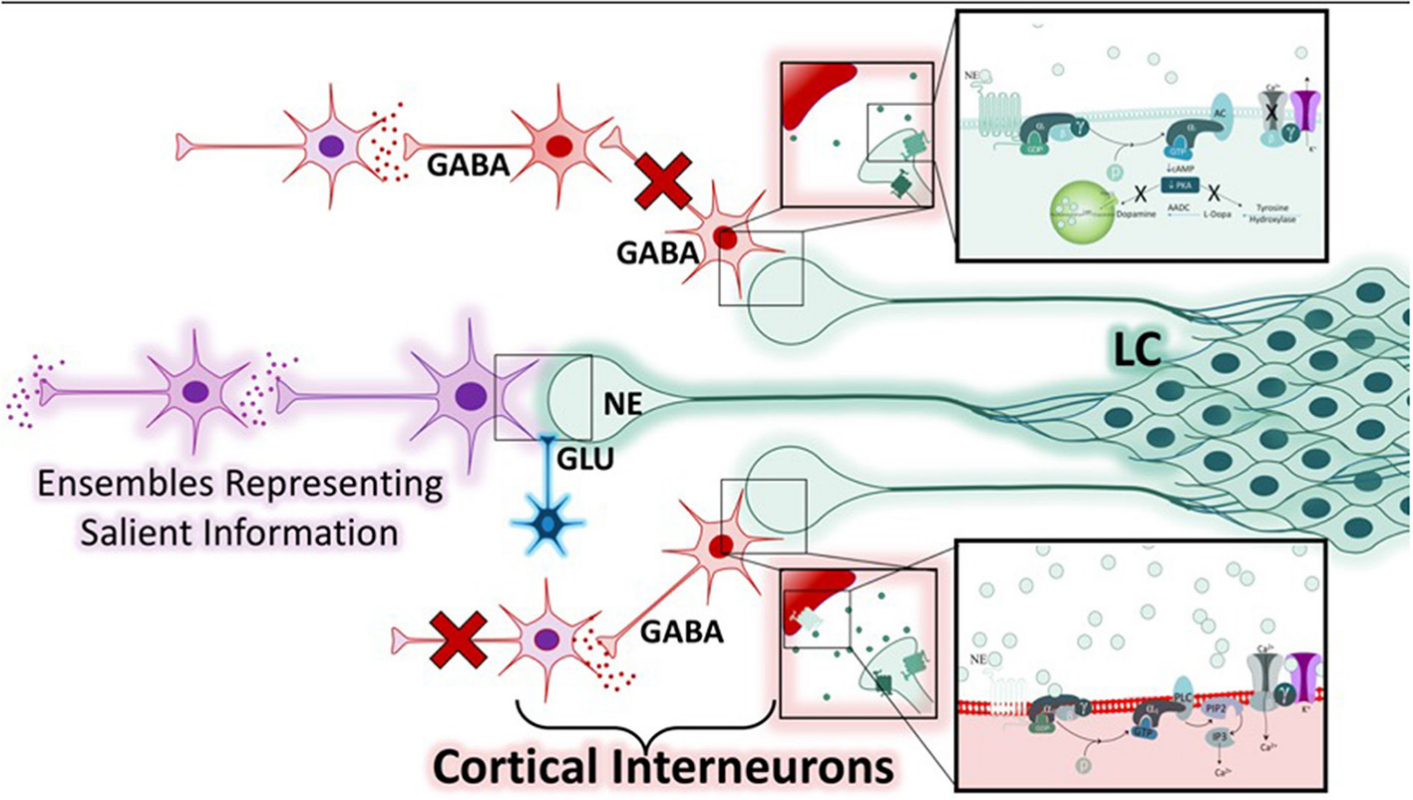
Mechanisms of norepinephrine-mediated lateral inhibition within the Glutamate Amplifies Noradrenergic Effects Framework. While neuronal ensembles transmitting prioritized information are expedited at noradrenergic hot spots (purple), regions containing low levels of norepinephrine and with low or absent glutamatergic input are suppressed. There are two mechanisms by which the noradrenergic system can accomplish this, both involve modulation of GABA-ergic interneurons (red). Depending on the adrenergic receptors expressed, and the concentration of norepinephrine at the synapse, various cellular responses may occur. Two examples are illustrated. The bottom neuronal path shows a cortical interneuron that expresses the α-1-adrenergic receptor (α1-AR) and is activated by moderate levels of norepinephrine. This results in the activation of the Gα_q_ in the post-synaptic cell, resulting in the depolarization of the GABAergic interneuron and inhibition of the neuronal path. The top neuronal path shows two GABA-ergic interneurons with feed-forward connections that are suppressed when pre-synaptic α2-adrenergic receptors are activated by low levels of norepinephrine, resulting in hyperpolarization and inhibition of neuronal firing. Thus, the varied affinity of the adrenergic receptors for norepinephrine, combined with the presence or absence of glutamate, is the cellular basis for this lateral-inhibition response, and ultimately serves to heighten the response of other neurons to salient information.

The second study, primarily based around the concept of arousal, is defined by Mather et al. as, a state “evoked by emotional events” that “enhances some aspects of perception and memory but impairs others” (6). The authors put forth the Glutamate Amplifies Noradrenergic Effects (GANE) theory (2016) to explain the ability of arousal to both enhance and suppress cognitive processes based on the priority of a stimulus. The GANE model proposes that arousal-induced NE release from the LC biases perception and memory to magnify the signal of salient, high priority neuronal ensembles while suppressing the signal of lower priority ensembles. In order to do so, the model proposes that the phasic activity of the LC, together with elevated levels of glutamate at the site of prioritized representations, increases NE release, creating “NE Hot Spots” (Figure 3). These regions are characterized by glutamate and NE co-release that advance the transmission of high priority neuronal ensembles (6). This excitatory effect is intensified by widespread NE-mediated suppression of weaker, low-priority neural responses via lateral and auto-inhibitory processes (6). This tenor is reminiscent of the 2015 report asserting that “attention” affects neuronal dynamics by altering the responsivity associated with a particular stimulus (5, 199), an effect thought to be mediated by interneuron-mediated lateral inhibition (Figure 4). Moreover, this mechanism is thought to normalize activity and suppress competing stimuli, thus resulting in an increased signal to noise ratio (5). Further, the authors suggest that these signals interact within the local cortical circuit to produce oscillatory synchrony so that “relevant representations” are selected and routed through the brain (5). Thus, a critical convergence in cognitive psychology, neuroscience, and neuroendocrinology bring together the concept that LC-NE mediated selective attention highlights salient stimuli, likely signaled by glutamate hotspots (6) and concurrently silencing irrelevant stimuli via lateral inhibitory mechanisms mediated by inhibitory interneurons (5, 6).

### Modules of Task-Specific Neuronal Sub-populations: Redefining LC-NE Function

Several recent methodological advancements in genetics and molecular biology have provided neuroscientists with tools to test direct causal hypotheses on neuroanatomical structure and function with exquisite temporal and spatial resolution. The application of intersectional genetics allows one to precisely target a specific cell type using a combination of enhancers and promoters unique to that cell type. The addition of an element for temporal control of the experimental manipulation, such as doxycycline-dependent t/TA or r/TA, allows for an even more powerful approach (200). Stemming from these technologies, genetic coding of microbial opsins that can be directed toward specific cell types with an enhancer-promoter approach enables neuroscientists to activate or inhibit neuronal activity with exposure to light (201). Such unprecedented advances have accelerated the study of neuroanatomy and physiology tremendously in recent years. Researchers using a novel optogenetic approach to study tonic and phasic activity in the LC, confirmed that the LC could fine-tune levels of arousal based on the frequency of its neuronal firing activity (202).

The concept of LC-NE mediated alterations in local cortical network interactions that ultimately dictate global brain states is supported by a study that demonstrates significant arousal induced, NE-dependent influence on cortical dynamics (203). The authors emphasize the potential role of ARs in mediating these effects, noting that low levels of NE during SWS and anesthesia preferentially recruit the high-affinity α-2 receptors coupled to inhibitory G proteins. In contrast, high NE levels during wakefulness may recruit low-affinity α-1 and β-ARs that couple to Gq or Gs proteins (203). More recent studies have examined the relationship between local desynchronization states and pupil-linked arousal in healthy human participants, providing intriguing evidence of LC-NE involvement in global network dynamic balance (204). The investigators used EEG recordings and pupillometry, while participants performed a challenging auditory discrimination task. EEG data from the auditory cortex were analyzed by a weighted permutation entropy algorithm that allowed investigators to capture not only features of desynchronization such as oscillatory power but also central underlying mechanisms detected as fluctuations in excitatory/inhibitory balance (204). This powerful approach elucidated two independent but synergistic processes that contribute to neural gain. First, local cortical desynchronization (EEG entropy) is dictated by regions of the sensory cortex concerned with task-related stimuli and is dependent on selective attention. Second, global brain states arise from processes related to pupil-linked arousal, a mechanism putatively dependent on LC-NE afferent projections. Together, the additive selective-attention mediated (local) gain and propagative arousal-mediated (global) gain optimize sensory perception (204).

An ongoing challenge emerges as we aspire to relate information about large-scale network dynamics on the scale of seconds to hours to the vast electrophysiological and neuroanatomical data measured on fast (sub-second) timescales (205, 206). A recent study approached this herculean task by measuring the activity of mPFC neuronal populations across 0.01–100 Hz frequencies using multi-site silicon probes (206). While slow timescale (global) dynamics were approximated by measuring a neuron's power spectrum on a global scale, fast timescale (local) dynamics that reflect the strength of a neuron's coupling within a population are approximated by frequency. The data, analyzed using frequency domain analysis, revealed no relationship in population neuronal coupling between time scales (i.e., neurons strongly coupled to a population on fast timescales could be weakly correlated to the same population on slow timescales). However, the results demonstrate a significant positive correlation between pupil-linked arousal and neuronal population dynamics at infraslow frequencies of 0.01–1 Hz (206). These findings are consistent with those of Waschke et al., as global, slow-timescale brain states were associated with LC-NE mediated pupil-linked arousal, while fast timescale, frequency-dependent local cortical dynamics, such as those involved in selective-attention within the sensory cortex, were not directly related. Thus, these analyses illustrate the breadth of responses of neuronal ensembles across fast and slow time scales (204, 206).

An excellent, recent review (207) highlights the LC-NE system as a prime candidate for such analyses. LC activation during attention orientation by salient somatosensory stimuli triggers rapid phasic LC responses offset by ~20 ms (110), while novel visual stimuli elicit a phasic LC response occurs over a timescale of 50–100 ms (118, 208, 209). Further, LC activity may also vary over a few seconds in working memory tasks, as well as over tens of seconds to minutes during cue-induced adjustments in behavioral task-related strategies (188, 210–218). These time-dependent processes have been studied using a combination of pupillometry, microdialysis, and single-unit recordings under conditions of pharmacological manipulation (207). The author also cites instances of behavioral states during learning (219), vigilance, and arousal that may vary over many minutes to hours (110, 118, 220, 221). Importantly, a recent study employing an optogenetic rodent model of LC activation was able to reproduce the temporal dynamics of stimulus-induced phasic LC activity for the first time (151). The study revealed that LC activation modulates cortical encoding of salience in a temporally and cell-type-specific manner in the somatosensory cortex. Two populations of LC-responsive cortical neurons could be distinguished; the first population was directly responsive to sensory detection in time with firing of the LC, while the second population was characterized by a lower basal firing rate and a “gating” long latency signal that only occurred after LC activation. Further, because the population of LC modulated cells could recruit the population of LC gated cells under phasic firing conditions, the authors hypothesize that these cortical cells were specialized to express highly salient information. Because the study exclusively tested photoactivation frequencies that do not induce arousal, these investigators delineated an arousal-independent role for LC in influencing cortical sensory processing, concluding that LC-induced attentional processing does not depend upon LC-NE–induced changes in arousal (151).

Emerging information on the LC-NE system indicates a growing need for computational tools. Until recently, the LC-NE system had been considered a homogenous nucleus of noradrenergic cells that fire synchronously and serve as a reactionary system that both centrally alerts and peripherally prepares for a behavioral response in the face of environmental threats to survival. However, accumulating data suggest that the LC may operate in a modular fashion, with specialized sub-regions that facilitate specific aspects of behavioral responses and cognition. The data are convincing, presenting anatomical, molecular, and electrophysiological evidence that at least two distinct populations of LC neurons exist that project to the prefrontal vs. motor cortex and are electrophysiologically and biochemically distinct (222). In support of this notion, a recent developmental genetic analysis study identified two subpopulations of LC neurons, a majority of which were derived from the alar plate, and a smaller portion, previously undescribed, is negative for a typical marker of alar-plate derived LC neurons (223).

### A Theory of Stress-Induced LC-NE Mediated Network Desynchronization

**Figure 5:**
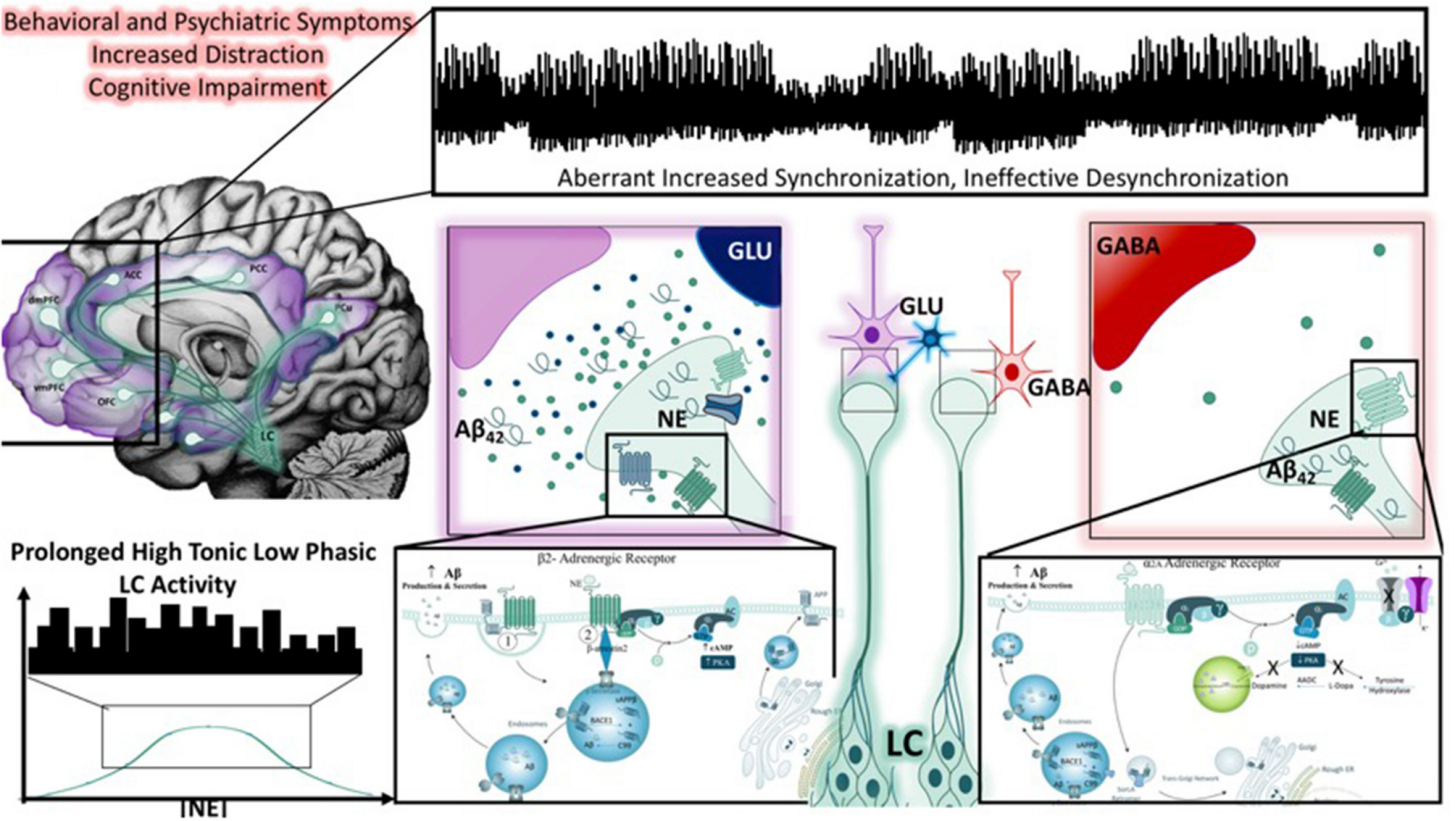
Noradrenergic modulation of Aβ_42_ peptide production and the impact of LC neuronal dysfunction. Behavioral and psychiatric symptoms resulting from global brain impairments have been associated with a number of stress-related disorders, including the dementia resulting from Alzheimer's disease. Throughout the literature, studies have demonstrated that cognitive impairment and epileptiform activity associated with dementia are associated with aberrant increased cortical synchronization, and reductions in desynchronization (illustrated in top panel). This may also reflect the inability of large-scale network switching between the default mode and salience networks, an activity thought to be heavily influenced by LC-NE activity (top left panel). One theory posits that chronic stress, marked by high tonic, low phasic LC activity (as depicted in the bottom left corner), promotes the accumulation of Aβ_42_ peptides LC neurons and results in neuronal injury. As depicted in the right panel, the adrenergic receptors that transmit or suppress information in a concentration-dependent, receptor-dependent fashion, are also known to influence the production of Aβ_42_ peptides. Prolonged periods of such activity, combined with aging could initiate LC neuronal degeneration. Importantly, in the presence or absence of LC degeneration, the impairment or low frequency of phasic firing may impact the transmission of salient information based on a flattening of the inverted U-curve on which the norepinephrine system operates. Salient information would be expected to be less quickly conveyed, and less important information may take longer to suppress.

Compelling pre-clinical (224, 225) and clinical evidence (226, 227) supports the involvement of the LC-NE system in Alzheimer's Disease (AD) via the influence of Ars (Figure 5) on the processing of amyloid precursor protein (APP) (228–230), dysregulation of the stress-signaling axis (231), and exacerbation of neuroinflammation (144). Throughout the lifetime, APP can undergo sequential β- and γ-secretase proteolytic processing to release a 42-amino acid length amyloid beta-peptide [Aβ_42_; (232)]. While Aβ_42_ is best known for its role as a self-nucleating peptide that readily forms concentration-dependent neurotoxic aggregates called plaques (233), it is now widely accepted that endogenous picomolar levels of Aβ_42_ are produced and efficiently broken down throughout the lifetime (234). However, the efficiency of Aβ_42_ metabolism is decreased with age [reviewed in (235)], causing an imbalance in the ratio of Aβ_42_/Aβ_40_, (236) resulting in the formation of amyloid plaques and initiating factors in a cascade of events that lead to neurodegeneration (237).

The work of Muresan and Muresan lay a critical, and potentially underappreciated, foundation for cellular mechanisms of APP processing and transport in brainstem neurons under normal and degenerative conditions *in vitro* (238, 239). Most pertinent to this review, a series of early studies that employ cultured LC-derived CAD cells suggests that the LC may be a region for Aβ_42_ plaque seeding, from which Aβ_42_ oligomers are released over the widespread terminal regions of the LC (240). Further investigation of a subpopulation of CAD cells that were prone to accumulatio of large amounts of intracellular Aβ_42_ at the terminals of their processes revealed significantly increased β-secretase immunoreactivity in noradrenergic axon terminals, that colocalized with Aβ_40, 42_ endosomal and autophagic marker immunoreactivity. The authors conclude that the initial seeds of aggregated Aβ_42_ are produced at the neurite terminals of LC neurons that project into the brain regions prone to AD pathology and these seeds then trigger further aggregation of soluble, extracellular Aβ_42_ into plaques, which are eventually exocytosed upon LC neuronal degeneration (240). In line with these findings, recent studies employing an unbiased, semi-quantitative stereotaxic neuropathology approach demonstrate significant changes in LC volume in AD post-mortem brains during various stages of disease progression (241), and suggests LC imaging may have biomarker potential for NE dysfunction in aging diseases (242).

From a large-scale circuit perspective, the Van Bockstaele lab put forth the similar hypothesis that chronic stress, or hyper-arousal-induced hyperactivation of the LC-NE system, would result in the aberrant production of endogenous Aβ_42_ over the course of decades; a dysfunction that would be relatively silent in the early stages of life but would contribute to cognitive dysfunction of dementia later in life [(243); Figure 5]. Fundamentally, our hypothesis was inspired by the work of Palop and Mucke, that emphasize a balance between inhibitory and excitatory networks for regular network synchronization and, therefore, cognitive functioning (244, 245). Palop and Mucke demonstrate that network desynchronization resulting from an imbalance in inhibitory and excitatory neurotransmission is a mechanism of cognitive disturbance in several mouse models of AD (245). Our group expanded on this notion by applying this circuit-perspective to the LC-NE system (243). In our review, we present evidence from the literature that the LC-NE system has a profound influence over glutamatergic (246) and GABAergic systems (247). Moreover, we suggest that a disturbance in the LC-NE system, caused by the deleterious effects of chronic stress and exacerbated by the presence of Aβ_42_, would be sufficient to induce the inhibitory/excitatory imbalance resulting in global network desynchronization (243). We emphasize the global architecture of the LC-NE system, its role in modulating the stress response, learning and memory, and inflammatory processes as unique features of the system that are also key disturbances in AD patient populations and post-mortem AD brain tissue (243).

Our laboratory is the first to have localized endogenous Aβ_42_ peptides to LC neurons of the naïve rat *in vivo* using fluorescence microscopy and immunoelectron microscopy (248). Using highly sensitive ELISA for Aβ_42_, we demonstrated that NE and Aβ_42_ levels positively correlate in the naïve rat. In mice null for the DBH gene and rats treated with the LC-selective neurotoxin DSP-4, we found decreased endogenous Aβ_42_ levels. Taken together with studies demonstrating β- and α- AR-mediated production of Aβ_42_, we believe that NE may play a role in modulating Aβ_42_ levels in the LC (248). This notion is supported by earlier pharmacological studies that indicate the activation of β-AR or α-AR can influence Aβ_42_ production, and that stimulation of β-AR on microglial cells can upregulate insulin-degrading enzyme, an enzyme responsible for the break-down of Aβ_42_ peptides (249). Thus, well-established mechanisms of NE-mediated Aβ_42_ production and degradation described in the literature would support our observations.

Subsequent studies in our lab examined the relationship between Aβ_42_ and the LC-NE system using a genetic model of chronic stress (250). The mice employed in these experiments conditionally overexpressed CRF (CRF-OE) in the forebrain upon administration of doxycycline (DOX) in their chow. Male and female DOX-treated mice were compared to their saline-treated littermates as the control. These studies revealed that CRF OE was sufficient to elicit a redistribution of Aβ_42_ peptides in LC somatodendritic processes in male and female mice without altering Aβ_42_ total protein levels. Under the electron microscope, lipofuscin, and abnormal morphology of lysosomal compartments were apparent, indicating that the compartments that usually clear Aβ_42_ peptides could have been worn down or become dysfunctional with CRF OE (250). We also observed swollen microvessels with lipid-laden vacuoles, a sign of blood-brain barrier dysfunction. Other potential injury signs were evident as CRF OE mice exhibited high glial astrocytic protein immunoreactivity (250).

While considering that hyperactivity of the LC, driven by chronic stress, could be deleterious to the long-term integrity of the LC-NE system, we sought to better understand mechanisms of LC regulation. There is evidence for LC auto-regulation by the release of NE from somatodendritic processes, but only when the firing rate is high (15–20 Hz), likely under phasic conditions (145, 251, 252). The high-frequency action potential most likely elevates residual calcium level in LC somata, which leads to NE release, as shown in chromaffin cells (253). NE is stored in central neurons within SSVs and LDCVs that are present in both cell somata and dendrites of LC neurons (55–57). Thus, we argue that NE somatodendritic release from LDCVs plays a protective role in maintaining LC-NE system integrity by preferentially activating α-2ARs coupled to inhibitory G proteins, therefore, decreasing the excitability of LC neuronal cell bodies.

In line with our hypothesis, a recently published theory presents a compelling argument for the impaired phasic discharge of LC neurons in neurodegenerative disease. Janitzky proposes that the presence of persistent high tonic discharge may impair the function and protective actions of phasic discharge (254). First, the conditions described by Janitzky are consistent with our hypothesis of stress-induced LC-NE dysregulation, as we would predict that LC-NE firing would be altered to reflect a “hyper-aroused” state, defined by high tonic and low phasic activity. Second, the idea that an impairment in phasic discharge could exacerbate the hyper-activity of the LC by preventing auto-regulatory mechanisms derived from LDCV somatodendritic release of NE and other neuropeptides is consistent with our hypothesized protective role of LDCV NE release. Further, the dysregulation of such mechanisms in stress-related disorders such as AD could contribute to LC neuronal degeneration (254). We continue to expand this hypothesis with a further inquiry into the behavioral and psychological symptoms of dementia, drawing lines of parallel between depression and AD. We put forth the notion that chronic stress is a factor that connects disparate aspects of both disorders and which profoundly alters LC-NE system integrity (255).

In the context of Mather and Harley's GANE hypothesis, the role of GABAergic interneurons is of particular interest. In this regard, the LC-NE system utilizes interneurons as lateral agents of inhibition to heighten the signal of salient information while minimizing the signal of irrelevant information. A study by the Mucke and Palop group (256) suggests that a voltage-gated sodium channel subunit Nav1.1, that is predominantly localized to parvocellular interneurons are responsible for the interneuron dysfunction at the root of pathological oscillatory rhythms and network synchrony in mouse models of AD (256). The study was conducted in cortical neurons via EEG, with a gamma frequency band reflecting the activity and function of such GABAergic interneurons. Interestingly, there is evidence of NE-regulation of parvocellular interneurons of the paraventricular nucleus of the hypothalamus (257, 258), as well as pyramidal interneurons of the cortex (259), although it is not specified if the interneurons are of the parvocellular subtype. The fascinating work of Dr. Tsai has elucidated a non-invasive method of induced 40 Hz gamma-band stimulation for the improvement of memory impairment and neuronal loss in AD by improving the clearance of Aβ_42_ plaques and hyperphosphorylated tau pathology in several mouse models of AD (260, 261). Thus, while it remains unclear if the LC-NE system is involved in this gamma oscillation related mechanism, there appears to be a promising rationale for further investigation.

Aside from accelerating the advancement of our understanding of the physiological properties of the brain, computational neuroscience is also a leading edge of modern neuroanatomy. The emergence of 3D EM reconstruction has allowed for the in-depth spatial, and morphological analysis of the brain microenvironment of healthy and pathological disease states that are complex and varied, such as in AD. One facet of AD pathology is an inflammatory state in which microglia and astrocytes surrounding affected neurons and Aβ_42_ plaques are activated—an event thought to exacerbate cognitive deficits in AD patients. Nuntagij et al. utilized 3D reconstruction EM in both 3 × Tg mice and naturally aged dogs to extensively describe the close spatial relationship between Aβ_42_ deposits and the neutrophil, revealing entangled and branched plaques engulf soma, and apical dendrites (262). The ability of 3D EM reconstruction to portray the complex, disruptive forces of Aβ plaques and neurofibrillary tangles are evident in Fiala et al. work, which describes swollen, dystrophic neurites within late-stage plaques that form pouches impaled with microtubules, forming loops that trap mitochondria and other organelles, rendering them non-functional (263). Thus, 3D EM analysis has been instrumental in establishing that intraneuronal Aβ_42_ aggregation may disrupt intracellular transport, leading to the dysfunction of mitochondria, potentially resulting in autophagic degeneration (264).

## Discussion

### Implications for Health and Disease

A recent study used structural equation modeling on an open-access dataset of magnetization transfer images from the Cambridge Center for Aging and Neuroscience cohort to test the hypothesis that LC signal intensity values would be more closely related to NE-dependent functions in older adults compared to younger adults. The investigators concluded that age-related reduction of LC structural integrity is associated with cognitive and behavioral impairments (265). In line with this, a study examining functional connectivity between the LC and SN in healthy young and older adults used regression and functional connectivity analyses on resting-state fMRI data over a time course of LC activity. The study provides evidence that older adults had reduced functional connectivity between the LC and SN compared with younger adults, evidenced by increased coupling of the CEN network to the SN than the DMN. These authors conclude that the reduced interactions between the LC and SN impair the ability to prioritize the importance of incoming events. In turn, the SN fails to initiate network switching (4, 176), which would promote further attentional processing (266). The continued study of open access data sets using computational tools such as multivariate analysis will increase the amount of information decoded from brain activity (267). In particular, the LC will be a fascinating subject of study over varied timescales and sub-regions, as it likely mediates the vast array of behavioral and physiological outputs by coding responses differentially over short and long timescales. A number of disorders arise from hyper-activation of the SN that directly or indirectly relates to dysfunction of the LC-NE system. Among them, a convincing case can be made for post-traumatic stress disorder and opiate use disorder, in which the aberrant assignment of high salience to trauma- or drug- experience related stimuli hinder patient progress to recovery (268).

### Future Directions

In light of the computational advancements discussed throughout this review, the expansive field of single-cell transcriptomics warrants mention here. Recent studies from the Allen Institute and others unveil a comprehensive view of genomic cortical neuron heterogeneity that cannot be overstated. The results predict the existence of at least 37 distinct peptidergic neuron types that are characterized by transcript abundance and taxonomic profiling, and compose cortical neuromodulatory networks (269). As we progress further into the age of transcriptomics, proteomics, and connectomics, a trend of increasing complexity in the form of neuronal heterogeneity emerges. While investigators continue to gather evidence of this diversity in evolutionarily conserved brain regions such as the LC, it is most apparent in brain regions that rapidly expanded in human evolution, such as the dl PFC. As pointed out by Arnsten et al. in response to Mather and Harley's 2016 GANE Theory (270), the dlPFC is a region associated with higher-order cognitive processing that is modulated in a specialized, often opposing, manner compared to the classic synapses of the sensory cortex, amygdala, and hippocampus (271). Thus, a critical future direction for LC-NE research will be in testing the GANE hypothesis in both classical and more newly evolved synapse types as they relate to neuronal health and disease. In this regard, leading studies have begun to examine transcriptomic profiles of the human dlPFC with the 10x genomics platform to uncover cortical layer-specific expression profiles (signatures), and promises to continually advance the notion of Psychiatric Genomics (272).

### Concluding Remarks

As we have moved forward, technological advancements in the evolving field of computational neuroscience have afforded scientists unprecedented resolution in the study of neural dynamics across spatial and temporal scales. In reference to the LC-NE system, this evolution has occurred across disciplines for decades. Today, with instruments of ever-increasing spatial and temporal resolution and data-driven analytics, the decades to come promise to reveal LC-NE system mechanics as they progressively unfold in space over time. The continued study of this multifaceted system in the context of stress- or arousal-related psychiatric disorders, including neurodegeneration, may facilitate a deeper understanding of within-network and between-network LC-NE dynamics. In time, undoubtedly, this will directly or indirectly lead to the discovery of novel therapeutics to treat the underlying systemic brain imbalances that drive the symptoms and progression of a wide array of such disorders.

## Author Contributions

EV and JR conceived the idea. JR wrote the manuscript. EV checked the manuscript for accuracy. Both authors contributed to the article and approved the submitted version.

## Conflict of Interest

The authors declare that the research was conducted in the absence of any commercial or financial relationships that could be construed as a potential conflict of interest.

## Abbreviations

ACTH: adrenocorticotropin releasing hormone
aINS: anterior insula
AR: adrenergic receptor
BOLD: Blood Oxygen Level Dependent
CEN: Central Executive Network
CRF: Corticotropin-Releasing Factor
CRF-OE: overexpressed
CRF, CRFR1: CRF receptor 1
DAB: 3,3a-diaminobenzidine tetrahydrochloride
dACC: dorsal anterior cingulate cortex
dlPFC: dorsolateral prefrontal cortex
DMN: Default Mode Network
dMPFC: dorsal medial prefrontal cortex
DOX: doxycycline
dPFC: dorsal frontal cortex
dPPL: dorsal posterior parietal lobe
DREADDs: designer receptors exclusively activated by designer drugs
DβH: dopamine-beta-hydroxylase
FEF: Frontal Eye Field
GANE: Glutamate Amplifies Noradrenergic Effects
HRP: Horseradish Peroxidase
ICA: independent component analysis
ICNs: Intrinsic Connectivity Networks
IPL: inferior parietal lobe
LC: Locus Coeruleus
LDCV: Large Dense-Core Vesicle
lPPL: lateral posterior parietal lobe
LTC: lateral temporal cortex
NE: Norepinephrine
PACE: Probability Associated Community Estimation
PCC: posterior cingulate cortex
pCu: precuneus
PET: Positron Emission Topography
PLACE: Path Length Associated Community Estimation
RAS: reticular activating system
REM: rapid eye movement
SN: Salience Network
SSV: Small Synaptic Vesicle
SWS: slow-wave sleep
TEM: Transmission Electron Microscope
TH: Tyrosine Hydroxylase
TPL: temporoparietal lobe
VAN: Ventral Attention Network
vMPFC: ventral medial prefrontal cortex
vPFC: ventral frontal cortex.
